# Ergostane-Type Steroids from Korean Wild Mushroom *Xerula furfuracea* that Control Adipocyte and Osteoblast Differentiation

**DOI:** 10.4014/jmb.2006.06013

**Published:** 2020-08-15

**Authors:** Seoung Rak Lee, Jin Hee Choi, Rhim Ryoo, Jin-Chul Kim, Changhyun Pang, Seon-Hee Kim, Ki Hyun Kim

**Affiliations:** 1 School of Pharmacy, Sungkyunkwan University, Suwon 649, Republic of Korea; 2 Sungkyun Biotech Co., Ltd., Suwon 16419, Republic of Korea; 3 Special Forest Products Division, Forest Bioresources Department, National Institute of Forest Science, Suwon 1661, Republic of Korea; 4 KIST Gangneung Institute of Natural Products, Natural Product Informatics Research Center, Gangneung 2551, Republic of Korea; 5 School of Chemical Engineering, Sungkyunkwan University, Suwon 16419, Republic of Korea

**Keywords:** *Xerula furfuracea*, Physalacriaceae, ergostane-type steroids, adipogenesis, osteogenesis

## Abstract

As part of our current work to discover structurally and/or biologically novel compounds from Korean wild mushrooms, we isolated five ergostane-type steroids (**1**-**5**) from the fruiting bodies of *Xerula furfuracea* via repeated column chromatographic separations and HPLC purification. The chemical structures of the isolated steroids were shown to be (22E,24*R*)-24-methylcholesta-4,22- diene-3,6-dione (1), ergosta-7,22-diene-3*β*,5*α*,6*β*-triol (2), ergosta-7,22-diene-3*β*,5*α*,6*β*,9*α*-tetraol (3), (22E,24*R*)-5*α*,8*α*-epidioxyergosta-6,22-diene-3*β*-ol-3-O-*β*-D-glucopyranoside (4), and (22E,24*R*)-5*α*,8*α*-epidioxyergosta-6,9,22-triene-3*β*-ol-3-O-*β*-D-glucopyranoside (5) based on comparison of the data regarding their spectroscopic and physical properties with those of previous studies. Notably, this is the first report on the presence of the identified steroids (**1**-**5**) in this mushroom. We tested compounds **1**–**5** to determine their effects on adipogenesis and osteogenesis in the mouse mesenchymal stem cell line C3H10T1/2 and found that compounds **4** and **5** suppressed the differentiation of stem cells into adipocytes. Notably, in addition to its suppressive effect on adipogenesis, compound **5** was also shown to promote the osteogenic differentiation of stem cells. These findings demonstrate that the bioactive compounds isolated might be effective for the treatment of menopause-associated syndromes, such as osteoporosis and obesity, as the isolated compounds were shown to suppress adipogenesis and/or promote osteogenesis of stem cells.

## Introduction

*Xerula furfuracea* (Peck) Redhead, Ginns, & Shoemaker (Physalacriaceae) is the most common *Xerula* species. It is synonymous with *Xerula radicata* (Relhan) Doerfelt or *Oudemansiella radicata* (Relhan) Singer, which is commonly known as the ‘deep root mushroom’ or the ‘rooting shank’ [1]. Previous studies have reported that this type of mushroom contains polysaccharides and primary metabolites, which exhibited useful pharmacological effects, including antioxidant, antiaging, immunomodulatory, antitumor, and antibacterial activities [2-5]. One of the most interesting molecules identified in a culture of this mushroom was oudenone, which was shown to have an inhibitory effect on tyrosine hydroxylase [6, 7]. Its biogenesis was speculated to be based on the synthesis as well as enzymatic cyclization of the open-chain hexaketide precursor into oudenone [6, 7]. However, despite having been investigated in several previous studies, few reports have described the bioactive fungal secondary metabolites from *X. furfuracea*.

As part of our continuous endeavor to discover structurally and/or biologically novel compounds from diverse natural sources, including wild mushrooms [8-16], we collected scores of endemic Korean wild mushrooms from mountainous areas during the hot and humid summer and prepared methanol (MeOH) extracts of them for chemical investigation. In this study, we used these various collected mushrooms to perform an extensive chemical analysis of the fruiting bodies of *X. furfuracea*. The column chromatographic separation of its MeOH extract and the subsequent high-performance liquid chromatography (HPLC) purification led to the isolation of five ergosterol derivatives (**1**-**5**), as shown via liquid chromatography/mass spectrometry (LC/MS)-based analysis. Their structures were elucidated by combining both the spectroscopic data and the results of the LC/MS analysis. To the best of our knowledge, this is the first time these five ergosterol derivatives (**1**-**5**) have been identified in *X. furfuracea*. The isolated compounds (**1**-**5**) were then tested to determine their effects on adipogenesis and osteogenesis in the mouse mesenchymal stem cell (MSC) line C3H10T1/2. Herein, we describe the isolation and structural characterization of the five ergosterol derivatives (**1**-**5**) as well as their potential effects on the reciprocal regulation of adipocyte and osteoblast differentiation.

## Materials and Methods

### General Experimental Procedures

The optical rotations were obtained using a Jasco P-1020 polarimeter (Jasco, USA). The nuclear magnetic resonance (NMR) spectra were obtained using a Varian UNITY INOVA 800 NMR spectrometer operating at 800 MHz (^1^H) and 200 MHz (^13^C), with chemical shifts given in ppm (δ). Preparative HPLC was performed using a Waters 1525 Binary HPLC pump with a Waters 996 Photodiode Array Detector (Waters Corporation, USA). Semi-preparative HPLC was conducted using a Shimadzu Prominence HPLC System with SPD-20A/20AV Series Prominence HPLC UV-Vis Detectors (Shimadzu, Japan). LC/MS analysis was carried out on an Agilent 1200 Series HPLC System (Agilent Technologies, USA) equipped with a diode array detector and a 6130 Series ESI mass spectrometer by using an analytical Kinetex column (4.6 × 100 mm, 3.5 μm). Precoated Merck silica gel F254 plates and RP-18 F254s plates were used for thin-layer chromatography (TLC). Spots were detected by TLC under UV light or by heating after spraying with anisaldehyde-sulfuric acid.

### Mushroom Materials

The fresh, fruiting bodies of *X. furfuracea* were collected at Gwacheon-si, Gyeonggi-do, Korea, in June 2015. A voucher specimen (SKKU-MGBB-2015-06) of the mushroom was authenticated by one of the authors (K.H.K.) and was deposited at the herbarium of the School of Pharmacy, Sungkyunkwan University, Korea.

### Extraction and Isolation

Completely dried *X. furfuracea* fruiting bodies (120 g) were extracted with 80% MeOH three times (2.0 L × 3) at room temperature and then filtered. The filtrate was subsequently evaporated in vacuo to obtain a crude MeOH extract (20 g). Then, this extract was dissolved in distilled water (700 ml), solvent partitioned with hexane, CH_2_Cl_2_, and EtOAc (each 700 ml × 3), and thereby we obtained 2.1 g, 1.8 g, and 1.1 g of extract, respectively. Through LC/MS analysis of each fraction, we found that the CH_2_Cl_2_-soluble fraction harbors several nonpolar compounds with simple UV patterns around 210-220 nm, which indicated the presence of steroids and/or terpenoids. Thus, the CH_2_Cl_2_-soluble fraction was loaded onto a silica gel (230–400 mesh) chromatography column and fractioned using a solvent gradient of CH_2_Cl_2_-MeOH (60:1-1:1, v/v) to give six eluted fractions (A-F). Six subfractions (C1-C6) were obtained from fraction C (72 mg) by preparative reversed-phase HPLC (Phenomenex Luna C18, 250 × 21.2 mm ID, 5 μm) using CH_3_CN-H_2_O (1:9–1:0, v/v, gradient system, flow rate: 5 ml/min). Subfraction C4 (10 mg) was purified via semi-preparative reversed-phase HPLC (Phenomenex Luna C18, 250 × 10.0 mm ID, 5 μm) with 80% MeOH/H_2_O (isocratic system, flow rate: 2 ml/min) to obtain compound **1** (4.2 mg, *t*_R_ = 27.0 min). Compounds **3** (2.5 mg, *t*_R_ = 35.0 min), **4** (4.7 mg, *t*_R_ = 37.5 min), and **5** (4.5 mg, *t*_R_ = 27.0 min) were isolated from subfraction C5 (19 mg) via semi-preparative reversed-phase HPLC (Phenomenex Luna C18, 250 × 10.0 mm ID, 5μm) with 90% MeOH/H_2_O (isocratic system, flow rate: 2 ml/min). Finally, subfraction C6 (14 mg) was separated via semi-preparative reversed-phase HPLC (Phenomenex Luna C18, 250 × 10.0 mm ID, 5 μm) with 90% MeOH/H_2_O (isocratic system, flow rate: 2 mL/min) to produce compound **2** (2.2 mg, *t*_R_ = 33.0 min).

**(22*E*,24*R*)-24-Methylcholesta-4,22-diene-3,6-dione (1)** – A white powder, [α]_25_^D^: +10.6° (*c* 0.09, CHCl_3_), electrospray ionization mass spectrometry (ESI-MS) *m/z*: 411.3 [M + H]^+^; ^1^H NMR (CDCl_3_, 800 MHz): δ 6.17 (^1^H, br s, H-4), 5.20 (^1^H, dd, *J* = 15.5, 7.0 Hz, H-23), 5.13 (^1^H, dd, *J* = 15.5, 7.5 Hz, H-22), 2.67 (^1^H, dd, *J* = 14.5, 3.5 Hz, H-7), 1.17 (3H, s, H-19), 1.03 (3H, d, *J* = 6.5 Hz, H-27), 0.91 (3H, d, *J* = 6.5 Hz, H-28), 0.83 (3H, d, *J* = 6.5 Hz, H-26), 0.81 (3H, d, *J* = 6.5 Hz, H-27), 0.72 (3H, s, H-18); ^13^C NMR (CDCl_3_, 200 MHz): δ 201.9 (C-6), 199.1 (C-3), 160.8 (C-5), 135.4 (C-22), 132.6 (C-23), 125.2 (C-4), 56.4 (C-17), 55.8 (C-14), 50.8 (C-9), 46.6 (C-7), 42.9 (C-24), 42.3 (C-13), 40.7 (C-20), 39.3 (C-12), 39.0 (C-10), 35.5 (C-1), 34.0 (C-8), 33.8 (C-2), 33.2 (C-25), 27.8 (C-16), 23.6 (C-15), 21.0 (C-21), 20.7 (C-11), 20.1 (C-27), 19.8 (C-26), 17.7 (C-28), 17.3 (C-19), 11.7 (C-18).

**Ergosta-7,22-diene-3*β*,5*α*,6*β*-triol (2)** – A white powder, [α]_25_^D^: -17.1° (*c* 0.15, CHCl_3_), ESI-MS *m/z*: 431.3 [M+ H]+; ^1^H NMR (CDCl_3_, 800 MHz): δ 5.25 (^1^H, dd, *J* = 16.0, 8.0 Hz, H-23), 5.15 (^1^H, dd, *J* = 16.0, 7.5 Hz, H-22), 5.03 (^1^H, d, *J* = 1.5 Hz, H-7), 4.03 (^1^H, m, H-3), 3.97 (^1^H, br s, H-6), 1.03 (3H, d, *J* = 6.5 Hz, H-21), 0.98 (3H, s, H-19), 0.93 (3H, d, *J* = 7.0 Hz, H-28), 0.85 (3H, d, *J* = 7.0 Hz, H-26), 0.83 (3H, d, *J* = 7.0 Hz, H-27), 0.57 (3H, s, H-18); ^13^C NMR (CDCl_3_, 200 MHz): δ 141.5 (C-8), 136.2 (C-23), 132.1 (C-22), 120.5 (C-7), 76.1 (C-5), 74.2 (C-6), 67.6 (C-3), 56.1 (C-17), 55.2 (C-14), 43.7 (C-13), 43.0 (C-9), 42.0 (C24), 40.9 (C-20), 39.9 (C-4), 38.0 (C-12), 33.8 (C-10), 33.3 (C-25), 32.7 (C-1), 30.0 (C-2), 28.35 (C-16), 23.5 (C-15), 22.4 (C-11), 21.4 (C-27), 20.1 (C-26), 19.8 (C-21), 18.8 (C-19), 17.8 (C-28), 12.5 (C-18).

**Ergosta-7,22-diene-3*β*,5*α*,6*β*,9α-tetraol (3)** – A white powder, [α]_25_^D^: -7.8° (*c* 0.12, CHCl_3_), ESI-MS *m/z*: 447.3 [M + H]^+^; ^1^H NMR (CDCl_3_, 800 MHz): δ 5.32 (^1^H, dd, *J* = 5.5, 2.5 Hz, H-7), 5.20 (^1^H, dd, *J* = 15.5, 6.5 Hz, H-23), 5.15 (^1^H, dd, *J* = 15.5, 7.0 Hz, H-22), 4.00 (^1^H, m, H-3), 3.63 (^1^H, m, H-6), 1.08 (3H, s, H-19), 1.00 (3H, d, *J* = 6.5 Hz, H-21), 0.90 (3H, d, *J* = 6.5 Hz, H-28), 0.82 (3H, d, *J* = 6.5 Hz, H-26), 0.80 (3H, d, *J* = 6.5 Hz, H-28), 0.61 (3H, s, H-18); ^13^C NMR (CDCl_3_, 200 MHz): δ 143.0 (C-8), 135.5 (C-22), 132.0 (C-23), 119.6 (C-7), 77.4 (C-5), 74.7 (C-9), 72.6 (C-6), 67.0 (C-3), 55.2 (C-17), 50.6 (C-14), 47.9 (C-24), 43.6 (C-13), 40.4 (C-20), 40.2 (C-10), 39.4 (C-4), 35.1 (C-12), 33.0 (C-25), 30.3 (C-2), 28.2 (C-16), 27.9 (C-11), 26.9 (C-1), 22.9 (C-15), 21.4 (C-21), 20.9 (C-19), 19.8 (C-27), 19.5 (C-26), 17.4 (C-28), 11.6 (C-18).

**(22*E*,24*R*)-5*α*,8α-Epidioxyergosta-6,22-diene-3*β*-ol-3-*O*-*β*-D-glucopyranoside (4)** - An amorphous powder,[α]_25_^D^: +7.5° (*c* 0.12, MeOH), ESI-MS *m/z*: 613.3 [M + Na]^+^; ^1^H NMR (pyridine-*d_5_*, 800 MHz): δ 6.51 (^1^H, d, *J* = 8.5 Hz, H-7), 6.23 (^1^H, d, *J* = 8.5 Hz, H-6), 5.27 (^1^H, dd, *J* = 15.5, 7.5 Hz, H-23), 5.19 (^1^H, dd, *J* = 15.5, 7.5 Hz, H-22), 4.46 (^1^H, m, H-3), 1.03 (3H, d, *J* = 6.5 Hz, H-21), 0.97 (3H, d, *J* = 6.5 Hz, H-28), 0.88 (3H, d, *J* = 6.5 Hz, H-27), 0.87 (3H, d, *J* = 6.5 Hz, H-26), 0.79 (3H, s, H-19), 0.77 (3H, s, H-18), Glc: δ 4.94 (^1^H, d, *J* = 7.5 Hz, H-1'), 4.50 (^1^H, dd, *J* = 12.0, 2.5 Hz, H-6'b), 4.41 (^1^H, dd, *J* = 12.0, 5.0 Hz, H-6'a), 3.86 (^1^H, ddd, *J* = 9.5, 5.0, 2.5 Hz, H-5'), 4.32 (^1^H, dd, *J* = 9.5, 8.5 Hz, H-4'), 4.25 (^1^H, dd, *J* = 9.0, 8.5 Hz, H-3'), 4.05 (^1^H, dd, *J* = 9.0, 7.5 Hz, H-2'); ^13^C NMR (pyridine-*d_5_*, 200 MHz): δ 136.1 (C-6), 136.0 (C-22), 132.3 (C-23), 131.0 (C-7), 103.0 (C-1'), 82.0 (C-5), 79.3 (C-8), 78.6 (C-5'), 78.3 (C-3'), 75.3 (C-2'), 73.8 (C-3), 71.5 (C-4'), 62.7 (C-6'), 56.3 (C-17), 52.0 (C-14), 51.8 (C-9), 44.7 (C-13), 43.0 (C-24), 40.1 (C-20), 39.5 (C-12), 37.4 (C-10), 35.1 (C-1), 34.6 (C-4), 33.3 (C-25), 29.0 (C-2), 29.0 (C-16), 23.6 (C-15), 21.1 (C-11), 21.1 (C-21), 20.2 (C-26), 19.9 (C-27), 18.1 (C-19), 17.9 (C-28), 13.0 (C-18).

**(22*E*,24*R*)-5*α*,8α-Epidioxyergosta-6,9,22-triene-3*β*-ol-3-*O*-*β*-D-glucopyranoside (5)** - An amorphous powder,[α]_25_^D^: +5.6° (*c* 0.11, MeOH), ESI-MS *m/z*: 611.3 [M + Na]^+^; ^1^H NMR (pyridine-*d_5_*, 800 MHz): δ 6.67 (^1^H, d, *J* = 8.5 Hz, H-7), 6.31 (^1^H, d, *J* = 8.5 Hz, H-6), 5.44 (^1^H, dd, *J* = 6.0, 1.9 Hz, H-11), 5.26 (^1^H, dd, *J* = 15.4, 7.7 Hz, H-23), 5.17 (^1^H, dd, *J* = 15.4, 8.5 Hz, H-22), 4.49 (^1^H, m, H-3), 1.05 (3H, s, H-19), 0.99 (3H, d, *J* = 6.6 Hz, H-21), 0.95 (3H, d, *J* = 6.9 Hz, H-28), 0.86 (6H, d, *J* = 6.8 Hz, H-26, H-27), 0.74 (3H, s, H-18), Glc: δ 4.93 (^1^H, d, *J* = 7.7 Hz, H-1'), 4.46 (^1^H, dd, *J* = 11.9, 2.5 Hz, H-6'b), 4.40 (^1^H, dd, *J* = 11.9, 4.7 Hz, H-6'a), 4.31 (^1^H, dd, *J* = 9.3, 8.8 Hz, H-4'), 4.24 (^1^H, dd, *J* = 9.0, 8.8 Hz, H-3'), 4.04 (^1^H, dd, *J* = 9.0, 7.7 Hz, H-2'), 3.84 (^1^H, ddd, *J* = 9.3, 4.7, 2.5 Hz, H-5'); ^13^C NMR (pyridine-*d_5_*, 200 MHz): δ 143.7 (C-9), 136.1 (C-23), 135.2 (C-6), 132.4 (C-22), 131.0 (C-7), 119.3 (C-11), 103.0 (C-1'), 82.7 (C-5), 78.6 (C-3'), 78.4 (C-8), 78.3 (C-5'), 75.3 (C-2'), 73.7 (C-3), 71.5 (C-4'), 62.6 (C-6'), 55.9 (C-17), 48.6 (C-14), 43.8 (C-13), 43.0 (C-24), 41.3 (C-12), 40.2 (C-20), 38.5 (C-10), 33.8 (C-4), 33.3 (C-25), 33.0 (C-1), 30.0 (C-2), 29.0 (C-16), 25,4 (C-19), 21.3 (C-15), 20.9 (C-21), 20.1 (C-27), 19.8 (C-26), 17.8 (C-28), 13.1 (C-18).

### Cell Culture

The C3H10T1/2 cell line, which originated from mouse embryonic fibroblasts, was cultured in Dulbecco’s Modified Eagle’s Medium supplemented with 10% heat-inactivated fetal bovine serum, 100 U/ml of penicillin, and 100 μg/ml of streptomycin at 37°C with 5% CO_2_. To measure adipogenic activity, C3H10T1/2 cells were plated in a 6-well plate at a density of 5 × 105 cells/ml. The cells were then treated with 1 μM of dexamethasone (DMS), 10 μM of troglitazone, 5 μg/ml of insulin, and 0.5 mM of 3-isobutyl-1 methylxanthine for 48 h. Subsequently, the cells were cultured for an additional 72 h with 10 μM of troglitazone and 5 μg/ml of insulin. To measure osteoblastic activity, C3H10T1/2 cells were plated in a 6-well plate at a density of 5 × 10^5^ cells and treated with 10 mM of β-glycerophosphate and 50 μg/ml of ascorbic acid. The culture medium was changed every 72 h.

### Oil Red O Staining

Cultured cells were washed with phosphate-buffered saline and fixed in 10% neutral-buffered formalin for 1 h at room temperature. The cells were then stained with a 0.5% filtered Oil Red O stock solution (ORO; Sigma, USA) of 200 mg/40 ml of isopropanol. Afterwards, the cells were washed with distilled water three times. To evaluate the intracellular triglyceride content, the stained cells were resolved with 1 ml of isopropanol and then the absorbance was measured at a wavelength of 520 nm.

### Alkaline Phosphatase Staining

Cultured cells were washed with 2 mM of MgCl_2_ and then incubated with an alkaline phosphatase (ALP) buffer (100 mM of Tris-HCl, pH 9.5; 100 mM of NaCl; 10 mM of MgCl_2_; and 0.05% Tween-20) for 15 min. The cells were then incubated in an ALP buffer containing 0.4 mg/mL of nitro-blue tetrazolium (Sigma) and 0.2 g/ml of 5-bromo-4-chloro-3-indolyl phosphate (Sigma). After washing with 0.5 mM of ethylenediaminetetraacetic acid, the cells were fixed with 10% neutral-buffered formalin for 1 h.

### mRNA Isolation and Real-Time Polymerase Chain Reaction (PCR)

The RNA was isolated from the cells using the NucleoZOL reagent (NucleoZOL; Macherey-Nagel GmbH & Co., KG, Germany). Complementary DNA (cDNA) was then synthesized from 0.5 μg of total RNA using a ReverTraAce qPCR RT Master Mix Kit (FSQ-201; Toyobo, Japan) with random primers. The synthesized cDNA was mixed with the amplification mixture containing the Thunderbird SYBR qPCR Mix (Toyobo) and primers. The cDNA was then subjected to 40 PCR amplification cycles using a Thermal Cycler Dice (Takara, Japan) and the results were normalized using the expression of 36b4. The primers used in this study are listed in Table 1.

### Statistical Analysis

One-way analysis of variance was used to determine whether there were any statistically significant differences between the control group and the test group.

## Results and Discussions

### Isolation and Chemical Characterization of Compounds

**Fig. 1:**
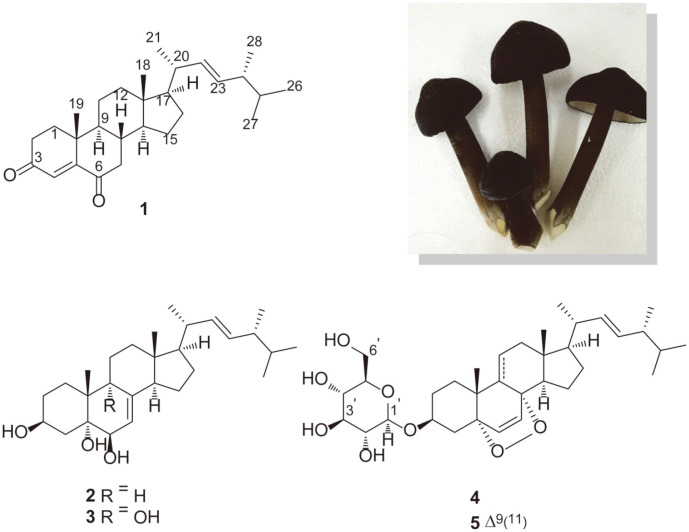
The chemical structures of compounds 1-5 and a representative image of the mushroom *X. furfuracea*.

The LC/MS-based chemical analysis of the methanolic extract of *X. furfuracea* fruiting bodies using sequential column chromatography [17], as well as the application of preparative and semi-preparative HPLC, resulted in the purification of five ergosterol derivatives (**1**-**5**) (Fig. 1). Compounds **1**-**5** were identified as (22*E*,24*R*)-24-methylcholesta-4,22-diene-3,6-dione (**1**) [18], ergosta-7,22-diene-3*β*,5*α*,6*β*-triol (**2**) [19], ergosta-7,22-diene-3*β*,5*α*,6*β*,9α-tetraol (**3**) [20], (22*E*,24*R*)-5*α*,8α-epidioxyergosta-6,22-diene-3*β*-ol-3-*O*-*β*-D-glucopyranoside (**4**)[21], and (22*E*,24*R*)-5*α*,8α-epidioxyergosta-6,9,22-triene-3*β*-ol-3-*O*-*β*-D-glucopyranoside (**5**) [22] by comparing their spectroscopic data with reported values from previously published studies. Compound **1** [(22*E*,24*R*)-24-methylcholesta-4,22-diene-3,6-dione] had been isolated and first reported from the marine sponge *Geodia cydonium* [18] in 1990, and previously, compound **2** (ergosta-7,22-diene-3*β*,5*α*,6*β*-triol) had been isolated from the endophytic fungus *Pichia guilliermondii* isolated from *Paris polyphylla* var. *yunnanensis* and exhibited weak antimicrobial activity against several bacteria [19]. Ergosta-7,22-diene-3*β*,5*α*,6*β*,9α-tetraol (**3**) was successfully obtained from the fruiting bodies of *Hygrophorus russula* where it showed cytotoxicity against both A549 and XF498 cell lines [20]. Compound **4** [(22*E*,24*R*)-5*α*,8α-epidioxyergosta-6,22-diene-3*β*-ol-3-*O*-*β*-D-glucopyranoside], which had been isolated from the mushroom, *Hericum erinacens* [21], was also obtained by us from the mushroom *Naematoloma fasciculare* in our previous study [23], where it was found to show cytotoxic activities against four human cancer cell lines (A549, SK-OV-3, SK-MEL-2, and HCT-15) [23]. Furthermore, (22*E*,24*R*)-5*α*,8α-epidioxyergosta-6,9,22-triene-3*β*-ol-3-*O*-*β*-D-glucopyranoside (**5**) was first isolated from the fruiting body of *Chlorophyllum molybdites* [22] and it showed moderate cytotoxicity against Kato III cells [22]. Therefore, based on a literature search and to the best of our knowledge, this is the first time these five ergostane-type steroids (**1**-**5**) were isolated and structurally elucidated from *X. furfuracea*.

### Regulatory Effects of the Compounds on the Differentiation of MSCs into Adipocytes and Osteoblasts

MSCs in the bone marrow are pluripotent cells known to differentiate into both osteocytes and adipocytes. Degenerative changes around the articular cartilage damages both the cartilage and bone, invading the subchondral bone and causing joint inflammation. Thus, the study of MSCs can help develop new treatments for joint damage. Recently, it was reported that adipose-derived stem/stromal cells (ASC) induced the differentiation of osteoblasts and that they could be used for the treatment of bone tissue damage [24]. Weight gain is a major post-menopausal phenomenon. After menopause, the conversion rate of cholesterol to estrogen decreases, blood cholesterol rises, and the amount of visceral fat in the fat tissue increases, resulting in increased abdominal obesity [25]. Moreover, as the inhibitory effect of estrogen on bone absorption disappears after menopause, a marked increase in bone loss occurs. In addition, osteoporosis significantly increases the risk of fractures in postmenopausal women [26]. Thus, it is necessary to develop a treatment that can control the differentiation of adipocytes and osteoblasts in order to improve menopausal complications, such as obesity and osteoporosis.

**Fig. 2:**
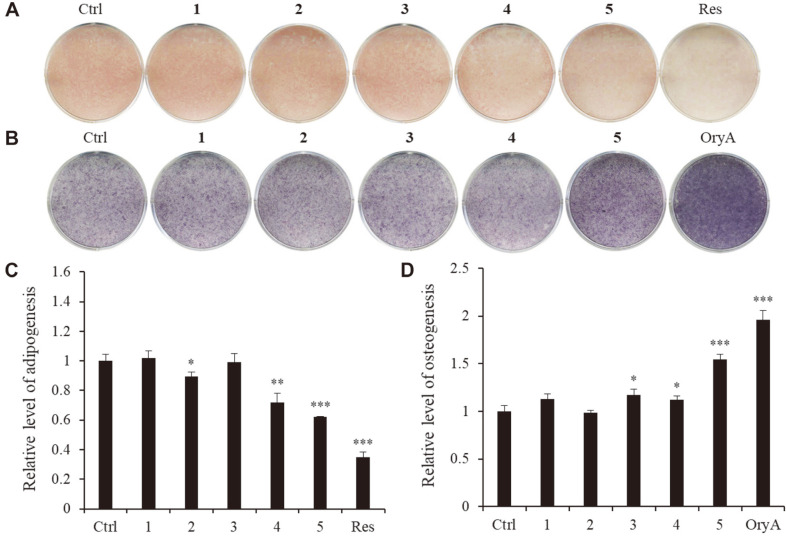
The effects of compounds 1–5 on the differentiation of MSCs into osteoblasts or adipocytes. The mouse MSC line C3H10T1/2 was treated with compounds **1**–**5**. After adipogenic differentiation, the cells were stained with ORO (**A**) and the stained cells were quantitatively evaluated by resolving the stained lipid droplets and measuring the absorbance at a wavelength corresponding to the red stain (**B**). In a separate plate, cells were differentiated into osteoblasts prior to ALP staining (**C**). Stained cells were quantitatively evaluated by measuring the absorbance at a wavelength corresponding to the blue stain (**D**). Ctrl represents the untreated negative control. A concentration of 20 μM of resveratrol (Res) was used as a positive control for adipogenesis. A concentration of 5 μM of oryzativol A (OryA) was used as a positive control for osteogenesis. The cells were treated with 10 μM of each of the compounds, which was added to the adipogenesis- or osteogenesis- differentiation medium. * denotes *p* < 0.05 and *** denotes *p* < 0.001.

Compounds **1**–**5** were tested for their effects on adipogenesis and osteogenesis in the mouse MSC line C3H10T1/2. When the cells were treated with compounds **1**–**5** during the adipogenic differentiation, compounds **4** and **5** were found to suppress the formation of lipid droplets when compared to the control cells, which were treated with a vehicle (Fig. 2). Compound **5** was also shown to have a stimulatory effect on osteogenic differentiation in MSCs. This indicates that compound **5** plays a dual role by suppressing adipogenesis while promoting osteogenesis.

### Effects of the Differentiation of MSCs into Adipocytes or Osteoblasts on mRNA Expression

**Fig. 3:**
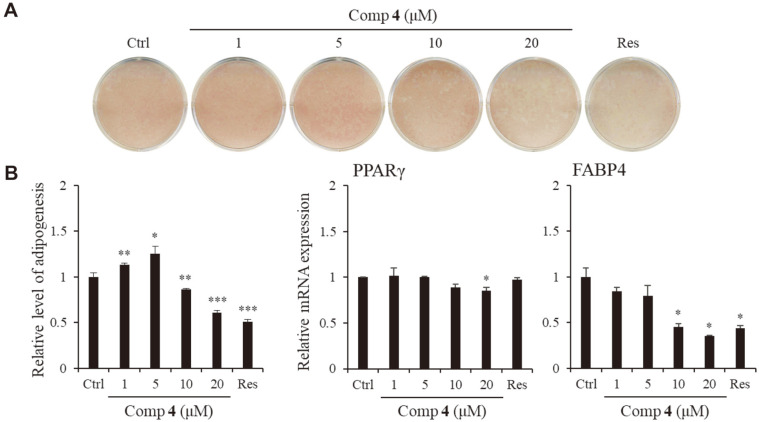
The suppressive effects of compound 4 on adipogenic differentiation. C3H10T1/2 cells were treated with sequential concentrations (1, 5, 10, and 20 μM) of compound **4** for 9 days prior to ORO staining (**A**). Stained cells were quantitatively evaluated by resolving the stained lipid droplets and measuring the absorbance at a wavelength corresponding to the red stain (**B** left). The expression of PPARγ (**B** center) and FABP4 (**B** right) mRNA was measured in compound 4-treated C3H10T1/2 cells using real-time PCR. A concentration of 20 μM of resveratrol (Res) was used as the positive control. * denotes 0.01 < *p* < 0.05, ** denotes 0.001 < *p* < 0.01, and *** denotes *p* < 0.001.

**Fig. 4:**
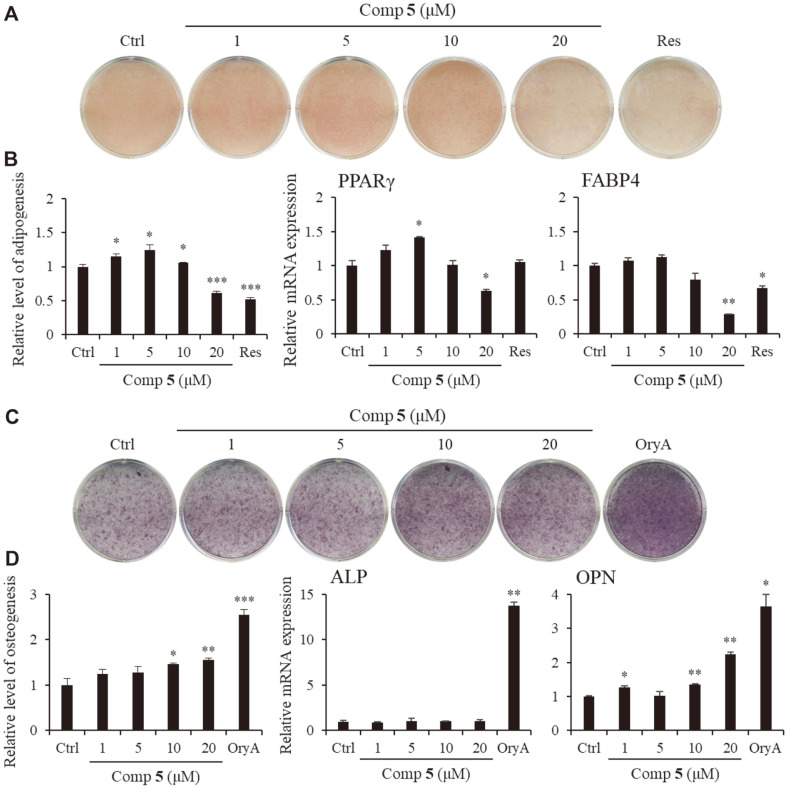
The effects of compound **5** on adipogenic and osteogenic differentiation. C3H10T1/2 cells were treated with sequential concentrations (1, 5, 10, and 20 μM) of compound **5** during adipogenic or osteogenic differentiation. The effects of compound **5** on adipogenesis were evaluated via ORO staining (**A**). Stained cells were quantitatively evaluated by resolving the stained lipid droplets and measuring the absorbance at a wavelength corresponding to the red stain (**B** left). The PPARγ (**B** center) and FABP4 (**B** right) mRNA expression were measured by real-time PCR. A concentration of 20 μM of resveratrol (Res) was used as the positive control. The activity of compound **5** on osteogenesis was evaluated via ALP staining (**C**). The stained cells were quantitatively evaluated by measuring the absorbance at a wavelength corresponding to the blue stain (**D** left). The ALP (**D** center) and OPN (**D** right) mRNA expression was measured by real-time PCR. A concentration of 5 μM of oryzativol A (OryA) was used as a positive osteogenesis control. * denotes 0.01 < *p* < 0.05, ** denotes 0.001 < *p* < 0.01, and *** denotes *p* < 0.001.

Compounds **4** and **5** demonstrated a suppressive effect on adipogenic differentiation in a dose-dependent manner (Figs. 3A and 4A). High doses of compounds **4** and **5** were found to reduce the mRNA expression of peroxisome proliferation-activated receptor γ (PPAR γ) and fatty acid-binding protein (FABP)-4, which are key factors of adipogenesis and lipid accumulation [27]. Both compounds **4** and **5** were shown to inhibit the differentiation of MSCs into adipocytes by suppressing the formation of lipid droplets and reducing the expression of adipogenesis-related genes.

Notably, in addition to its suppressive effect on adipogenesis, compound **5** was also shown to promote the osteogenic differentiation of MSCs (Fig. 4C). To test the effect of compound **5** on osteogenic differentiation, the cells were stained with ALP and quantitatively analyzed. Our results showed that increased concentrations of compound **5** led to the appearance of darker-colored cells, which demonstrates that the treated cells exhibited greater promotion of bone differentiation than the control group. Moreover, compound **5** enhanced the gene expression of osteopontin (OPN), which is an osteogenesis-related factor, during osteogenic differentiation in a dose-dependent manner (Fig. 4D) [28].

In conclusion, we identified five ergostane-type steroids (**1**-**5**) present in the fruiting bodies of *X. furfuracea* via LC/MS-based analysis. The identified steroids (**1**-**5**) were isolated for the first time from *X. furfuracea*. Ergostane-type steroids derived from various natural sources are gaining increasing attention due to their wide-ranging potential pharmacological activities including cytotoxic, antibacterial, hypocholesterolemic, neuritogenic, antifungal, anti-inflammatory, anti-allergic, and antioxidative activity [29, 30]. To date, a lot of data regarding the bioactive ergostane-type steroids has been investigated and accumulated [29, 30]. In this study, we examined the effects of the isolated compounds on MSC differentiation into adipocytes and osteoblasts. Two sterols out of five ergostane-type steroids, namely compounds **4** and **5**, were shown to inhibit the expression of genes involved in MSC differentiation toward adipocytes. Interestingly, compound **5**, which suppressed adipocyte differentiation, induced the differentiation of MSCs into osteoblasts, demonstrating a dual function. Therefore, this study revealed that (22*E*,24*R*)-5*α*,8α-epidioxyergosta-6,9,22-triene-3*β*-ol-3-*O*-*β*-D-glucopyranoside (**5**) isolated from the fruiting bodies of *X. furfuracea* plays two roles in MSC differentiation by promoting osteogenesis and inhibiting adipogenesis. Taken together, our results suggest that these bioactive compounds may be useful as an effective treatment for menopause-associated syndromes, such as osteoporosis and obesity.

## Figures and Tables

**Table 1:** The primers used for real-time polymerase chain reaction.

Acidic ribosomal phosphoprotein P0 (36b4)	forward	5′-AGATGCAGCAGATCCGCAT-3′
	reverse	5′-GTTCTTGCC- CATCAGCACC-3′
Peroxisome proliferator- activated receptor γ (PPARγ)	forward	5′-CCATTCTGGCCCACCAAC-3′
	reverse	5′-AATGCGAGTGGTCTTCCATCA-3′
Fatty acid binding protein 4 (FABp4)	forward	5′-CACCGCAGACGACAGGAAG-3′
	reverse	5′-GCACCTGCACCAGGGC-3′
Alkaline phosphatase (ALP)	forward	5′- CAAGGATGCTGGGAAGTCCG -3′
	reverse	5′- CGGATAACGAGATGCCACCA -3′
Osteopontin (OPN)	forward	5′- CTGGCAGCTCAGAGGAGAAG -3′
	reverse	5′- CAGCATTCTGTGGCGCAAG -3′
